# Supplementary material for: A novel haemocytometric COVID-19 prognostic score developed and validated in an observational multicentre European hospital-based study
Source: eLife. 2020 Nov 26;9:e63195. doi: 10.7554/eLife.63195 (PMC7732342; doi:10.7554/eLife.63195)
Supplement: Supplementary file 1. [file elife-63195-supp1.docx]

**Table S1**. **Basic demographic characteristics of COVID-19 PCR confirmed patients enrolled for prognostic score development by hospital**

| **Hospital** | 1 | 2 | 3 | 4 | 5 | 6 | 7 | 8 | 9 |
| --- | --- | --- | --- | --- | --- | --- | --- | --- | --- |
| All patients n [%] | 20 | 26 | 41 | 80 | 96 | 121 | 133 | 213 | 252 |
| Age Years (range) | 34 - 88 | 26 - 81 | 18 - 83 | 30 - 93 | 28 - 89 | 31 - 90 | 22 - 89 | 25 - 96 | 19 - 94 |
| Age Years (median) | 62.5 | 62.5 | 62 | 69 | 68 | 69 | 72 | 69 | 75.5 |
| **Females** |  |  |  |  |  |  |  |  |  |
| n [%] | 7 (35%) | 7 (26.9%) | 14 (34.1%) | 29 (36.2%) | 33 (34.4%) | 47 (38.8%) | 46 (34.6%) | 50 (23.5%) | 81 (32.1%) |
| Age Years (range) | 39 - 74 | 38 - 71 | 18 - 76 | 41 - 93 | 28 - 87 | 40 - 90 | 22 - 89 | 25 - 95 | 19 - 94 |
| Age Years (median) | 64 | 56 | 62 | 71 | 68 | 67 | 74 | 71.5 | 73 |
| **Males** |  |  |  |  |  |  |  |  |  |
| n [%] | 13 (65%) | 19 (73.1%) | 27 (65.9%) | 51 (63.8%) | 63 (65.6%) | 74 (61.2%) | 87 (65.4%) | 163 (76.5%) | 171 (67.9%) |
| Age Years (range) | 34 - 88 | 26 - 81 | 42 - 83 | 30 - 87 | 35 - 89 | 31 - 90 | 35 - 89 | 32 - 96 | 30 - 93 |
| Age Years (median) | 62 | 66 | 66 | 67 | 68 | 72 | 71 | 68 | 77 |
| **Length of hospitalisation** |  |  |  |  |  |  |  |  |  |
| Days (range) | 1 - 58 | 2 - 44 | 0 - 78 | 0 - 31 | 0 - 38 | 1 - 30 | 0 - 31 | 0 - 46 | 0 - 70 |
| Days (median) | 14.5 | 9 | 15.5 | 7 | 7 | 5 | 7 | 6 | 5 |
| **Comorbidities** |  |  |  |  |  |  |  |  |  |
| Absent [%] | 25% | 27% | 24% | 44% | NA | NA | 47% | 33.3% | 31% |
| Present [%] | 75% | 73% | 76% | 56% | NA | NA | 53% | 66.7% | 69% |
| **Disease severity** |  |  |  |  |  |  |  |  |  |
| Mild [%] | 0% | 0% | 10% | 0% | 9% | 0% | 4% | 13% | 8% |
| Moderate [%] | 20% | 8% | 7% | 20% | 25% | 39% | 22% | 9% | 21% |
| Severe [%] | 30% | 46% | 27% | 45% | 32% | 25% | 38% | 38% | 25% |
| Critical [%] | 35% | 27% | 49% | 19% | 20% | 11% | 8% | 8% | 14% |
| Fatal [%] | 15% | 19% | 7% | 16% | 14% | 25% | 28% | 32% | 32% |

Note: NA= not available
