# Supplementary material for: A novel haemocytometric COVID-19 prognostic score developed and validated in an observational multicentre European hospital-based study
Source: eLife. 2020 Nov 26;9:e63195. doi: 10.7554/eLife.63195 (PMC7732342; doi:10.7554/eLife.63195)
Supplement: Supplementary file 2. [file elife-63195-supp2.docx]

**Table S2. Novel parameters of different manufacturers in relation to possible adaptability of the haemocytometric COVID-19 prognostic score to non-Sysmex haematology analysers**

| **Company** | **Sysmex** | **Abbott** | **Beckman Coulter** | **Horiba** | **Mindray** | **Siemens Healthineers** |
| --- | --- | --- | --- | --- | --- | --- |
| Models | XN-Series | CELL-DYN Sapphire  Alinity hq | UniCel DxH800 /900  UniCel DxH600 /690T | Yumizen H1500, H2500 | BC-6800,  BC-6000, BC-6200, BC-6800+ | Advia 2120i  Advia 120 |
| White blood cell parameters | | | | | | |
| Immature granulocytes | IG %, # | IG %, # | *EGC %, #*  *(Early granulocytic cell)* | IMG %, IMG #  (Immature granulocyte) | IMG  (Immature granulocyte)  *IME %, #*  *(Immature Eo)* | DNI  (Delta neutrophil index) |
| Neutrophil parameters | NEUT-RI  NEUT-GI  (reactivity & granularity index) |  | *Mean neutrophil volume (MNV), neutrophil distribution width (NDW)* |  |  | DNI (delta neutrophil index) |
| Lymphocyte parameters | AS-LYMP %, #  RE-LYMP %, #  (Antibody synthesising lymphocytes, Reactive lymphocytes) |  |  | IML %, IML #  ALY#, ALY%  (Immature lymphocyte, atypical lymphocyte) | HFC %, #  (high fluorescent cells e.g. atypical lymphocytes or blasts) |  |
| Monocyte parameters | *Re-Mono*  *(Reactive monocytes)* |  | MDW (ESId)  (Monocyte distribution width/Early sepsis indicator) | IMM %, IMM #  (Immature monocyte) |  |  |
| Other WBC parameters |  |  |  | LIC %, #  (Large immature cells) |  | LUC  (Large Unstained Cells) |

**Table S2 continued.**

| Company | Sysmex | Abbott | Beckman Coulter | Horiba | Mindray | Siemens Healthineers |
| --- | --- | --- | --- | --- | --- | --- |
| Red blood cell parameters | | | | | | |
| Reticulocyte haemoglobin | RET-He  (reticulocyte haemoglobin equivalent) | MCVr (Mean cellular volume of reticulocytes)  *MCHr (mean cellular hemo-globin of reticulocytes)*  *CHCr (mean cellular hemo-globin concentration of reticulocytes)* | *MRV (mean reticulocyte volume)* | RHCc (Reticulocyte Hemoglobin  Content calculated)  MRV (Mean Reticulocyte Volume) | RHE (Reticulocyte Hemoglobin Expression)  *MRV (mean reticulocyte volume)*  *MVCr (reticulocyte average volume)* | CHr (Content Hemoglobin reticulocytes) |
| Nucleated red blood cells | NRBC %, # | NRBC %, # | NRBC %, # | NRBC %, # | NRBC %, # | NRBC %, # |
| Other RBC parameters | Hypo-He (%)  (hypochromic RBC)  RBC-He  (RBC haemoglobin content)  Delta-He  (difference between RET-He and RBC-He)  Micro% (microcytic RBC%) | %HPO (%)  (hypochromic cells) | *LHD (%)*  *(Low hemoglobin density)*  *MAF (Microcytic anemia factor)*  *RSF (RBC size factor)*  *MRV (mean reticulocyte volume)* |  | Hypo%  (Hypochromic RBC%)  *Micro%*  *(Microcytic RBC%)* | Hypo (%)  (hypochromic RBC) |
| Platelet parameters | | | | | | |
| Immature platelet fraction | IPF %  IPF # | %rP  (percentage of reticulated platelets) |  |  | IPF % | %Rtc Plts  Rtc Plts Count  (reticulated platelets %, #) |

Note: Recent technological advancements in haematology analysers have produced many novel parameters to characterise blood cells (Ref). While some are common, each manufacturer has parameters exclusive to their technology. The prognostic score could thus be adapted and trialled by using alternative parameters that provide similar assessment of cellular activation status, which is a core contributor to the discriminatory power of the prognostic score. This table is not meant to reflect a comprehensive list of all non-standard parameters available on all models of haematology analysers from the manufacturers listed here. The information shared here is limited to those parameter classes that in the authors opinion best match the parameters that make up the haemocytometric COVID-19-score. Parameters in black are diagnostic parameters, parameters in blue and in italics are research parameters. The information provided in this table was extracted from the latest publicly accessible version of instructions for use of the analysers mentioned. Whilst every effort was made to ensure that the data is correct, the authors do not take responsibility for any omissions or mistakes.

Ref. Rastogi P, Bhatia P, Varma N. Novel Automated Hematology Parameters in Clinical Pediatric Practice. Indian Pediatr. 2017;54(5):395-401.
